# Efficacy of a Voluntary Self-exclusion Reinstatement Tutorial for Problem Gamblers

**DOI:** 10.1007/s10899-021-09998-x

**Published:** 2021-01-23

**Authors:** Nigel E. Turner, Jing Shi, Janine Robinson, Steve McAvoy, Sherald Sanchez

**Affiliations:** 1 Centre for Addiction and Mental Health, Institute for Mental Health Policy Research, 33 Russell Street, Toronto, ON M5S 2S1 Canada; 2 Dalla Lana School of Public Health, University of Toronto, Toronto, ON Canada; 3 School of Rehabilitation Science, McMaster University, Hamilton, ON Canada; 4 The Responsible Gambling Council of Ontario, Toronto, Canada

**Keywords:** Self-exclusion, Problem gambling, Pathological gambling, Disordered gambling, Gambling intervention, Self-exclusion

## Abstract

Voluntary self-exclusion programs allow gamblers to voluntarily be denied access to gambling venues for an agreed upon period. Many people who self-exclude decide to return to gambling venues after the exclusion period has ended, however people who reinstate may be at risk for the recurrence of gambling problems. This study was designed to determine the efficacy of a tutorial created with the intent of reducing the risk of harm to those who reinstate. People who wished to be reinstated were asked to complete a survey on gambling related issues and then watch the tutorial video. An online video-based tutorial designed to reduce gambling related harm and to provide information about treatment services was developed. The control group (N = 131) consisted of people who reinstated in the year prior to the implementation of the online tutorial. The experimental intervention group (N = 104) were those who reinstated after the implementation of the online tutorial. There was a significant decrease in gambling and problem gambling comparing pre-exclusion to during exclusion in both the experimental and control group. Furthermore, this drop in gambling problem was sustained for 6-months and 12-months after reinstatement. However, no main effect or interaction was found that supported the efficacy of the tutorial. Self-exclusion by itself was associated with a sustained reduction in problem gambling. There was no significant evidence that the educational tutorial had any additional impact on the reinstatement process.

**Supplementary Information:**

The online version contains supplementary material available at (10.1007/s10899-021-09998-x).

## Introduction

Voluntary self-exclusion is a self-directed intervention offered by many gambling venues such as casinos that can be used by gamblers who want to ban themselves from access to the gambling venue (Nower and Blaszczynski 2008a) or from accessing online gambling websites (Luquiens et al., 2018). This self-directed intervention is often the first serious attempt a person makes to control their gambling (Blaszczynski et al. 2004). Periods of self-exclusion vary, with some programs placing a permanent ban on the individual and others offering the possibility of reinstatement after a period from one day up to several years. At the end of a self-exclusion period, some individuals choose to remove their self-exclusion and reinstate to re-gain access to the gambling venue. Very little research has focused on best practices for reinstatement after self-exclusion.

There is evidence that self-exclusion can be an effective means of encouraging responsible gambling (Gainsbury 2014; Luquiens et al. 2018; Nowatzki and Williams 2002). The type of individuals who self-exclude ranges from young adults to seniors and the strategy appears to be used approximately evenly by males and females. Motives for individuals to self-exclude have been found to be linked to a fear of suicide (Nower and Blaszczynski 2008b), internal motives related to an issue of addiction, and external motives related to characteristics of the gambling venue itself (Luquiens et al. 2018). Self-excluders have generally been found to be heavy gamblers, with many of them meeting the DSM-5 criteria for gambling disorder (Lhommeau et al. 2015; Luquiens et al. 2018). Lhommeau et al. (2015) found that no self-excluding participants in their study had a gambling treatment history, which is especially concerning given the prevalence of gambling disorders found among the group. Due to the high rates of self-excluders using self-exclusion programs multiple times, it has been suggested that additional protective measures such has treatment referrals or improved coordination between gambling venues and treatment clinics be put in place (Lhommeau et al. 2015; Luquiens et al. 2018). Furthermore, many problem gamblers change their minds about self-exclusion and wish to return to gambling (Gainsbury 2014; Nowatzki and Williams 2002). As such, it would be helpful to have validated resources designed to decrease the likelihood of them returning to problematic levels of gambling after reinstatement (Gainsbury 2014; Nowatzki and Williams 2002).

At the end of a self-exclusion period, some individuals choose to remove their self-exclusion and reinstate to re-gain access to the gambling venue. Reinstatement can be passive where the customer can automatically re-enter the casino after the end of the exclusion period, or active where the customer has to apply to be reinstated (Responsible_Gambling_Council 2016).There is currently limited research into the best practices for renewal and reinstatement (Canadian Partnership for Responsible Gambling 2018; Price 2016; Responsible_Gambling_Council 2016). According to Price (2016) best practice guidelines recommend mandatory services for high risk gamblers at the time of reinstatement such as a safe gambling plan, a brief educational course (online or in-person), or professional counselling (Price 2016). To the best of our knowledge, there has not been any empirical studies that evaluate the effectiveness of mandatory services during the reinstatement process, particularly for an educational course. The purpose of this research was to examine the reinstatement process and to evaluate the efficacy of an interactive reinstatement video tutorial (educational course) aimed at reducing problematic gambling among self-excluders who choose to re-engage in gambling activities. Five hypotheses guided this study. At 6 and 12-months after reinstatement:

### H1

Self-exclusion will reduce problematic gambling.

### H2

People in the experimental (tutorial) condition will be gambling less often and less problematically than those in the control group (no tutorial).

### H3

People in the experimental group will be more likely to seek treatment or return to self-exclusion than those in the control condition.

### H4

People in the experimental condition will have higher scores on the knowledge test than those in the control group.

### H5

People in the experimental group will be less likely to express a strong desire to gamble than those in the control group.

## Methods

### Participants

A total of 235 people completed the initial survey (59% male). The control group was recruited from May 2015 to April 2016 before the implementation of the reinstatement tutorial video (n = 131), and the experimental group was recruited from May 2016 to May 2017 after the implementation of the reinstatement tutorial video (n = 104). See Table 1 for sample characteristics. 107 participants completed the 6-month follow-up survey, and 112 participants completed the 12-month follow-up survey. For the follow up data, 107 participants completed the questionnaire at 6-month and 113 participants completed the questionnaire at 12-months. For the repeated measures analyses for those who only completed the 6-month follow-up survey, we substituted the 6-month data for the 12-month data and vice versa so that provide us with 130 complete records of follow up data. In addition, there was one missing value for the PGSI during self-exclusion; this was replaced with the pre-exclusion value.

**Table 1:** Participant characteristics (N = 235)

	n = 235	(%)
Sex	Male	59.0
Completion rate	6-months	45.0
	12-months	47.0
Age	19–25	9.4
	26–35	20.9
	36–45	21.4
	46–55	23.8
	56–65	18.0
	66 +	7.3
Marital Status	Married/common law	49.2
	Separated/divorced	16.2
	Single	30.2
	Widowed	3.2
Education	Completed College/University	55.1
	Completed secondary	29.9
	Some secondary	14.1
Income	> $40,000	31.2
	$40,000–$79,999	41.9
	$80,000–$119,999	16.2
	$120,000–$159,999	7.7
	> $160,000	2.7
Ethnic group	Caucasian	44.0
	Not caucasian	29.5
	Not answered	26.5

### Intervention

The Interactive reinstatement video tutorial was developed jointly by the OLG and problem gambling education staff at the Centre for Addiction and Mental Health, Toronto. The third author of this paper, JR, developed the content of the tutorial. The tutorial was designed to provide practical information to players who choose to be reinstated and return to gambling. The tutorial was an interactive video that consisted of information about gambling, harm reduction and counselling and also included a quiz with feedback to improve the participant’s knowledge of games and to help them stay in control while gambling (see Supplement text Table 1for an outline of the tutorial content). The online tutorial took 20–30 min to complete.

### Procedures

This study made use of a natural experiment as a sampling frame resulting from the implementation of the tutorial. People who self-excluded before the implementation of the reinstatement tutorial were the control group and people who reinstated after the implementation of the tutorial were the experimental group. In Ontario, Canada, reinstatement is active. The crown corporation which manages gambling in the provide, Ontario Lottery and Gaming Corporation (OLG), offers terms of 6 months, 12 months, and indefinite. Individuals are only eligible to apply for reinstatement after 6 months if they selected 6 months or indefinite self-exclusion, and after 12 months if they selected 12 months (Responsible Gambling Council, 2016). The reinstatement process was as follows:

1. To begin the process of reinstatement, the individual would have to submit a request in writing to a gaming site to be considered for reinstatement after the required amount of time has been completed.
2. The gaming site would then review the request and if eligible for reinstatement would send a letter to the player explaining the process.
  - For all participants in this study, the letter also included a brief description of the research study and a link to the online survey. They were also told that the survey was voluntary and that they would receive a $50 gift certificate for participating.
  - In the experimental group they were also given a link to the reinstatement tutorial that had to be completed before they could schedule a meeting with security staff at the gaming site.
3. The individual then scheduled a meeting at the gaming site which would occur no sooner than 30 days after the request to reinstate was received by the gaming site.
4. At the reinstatement meeting the individual would sign a form acknowledging their reinstatement and would be provided with a package of information and resources about positive play and support services.
5. Gaming staff would then remove the person’s exclusion status in its self-exclusion database (Ontario Lottery and Gaming Corporation, 2016).

The survey and the tutorial could be completed online or at the PlaySmart Centres in the casinos (formerly called Responsible Gambling Centres). The PlaySmart Centres provide players with information about the games, their odds, responsible gambling, information about treatment services, or provide an opportunity to simply take a break. PlaySmart information centres are located at all casinos in Ontario, and at the larger casinos the centres have staff trained in providing information about gambling, however most customers do not make use of the PlaySmart Centres. The only difference between the control group and the experimental group was that the experimental group was required to complete the online tutorial, whereas the control group simply received the package of information about responsible gambling.

Follow-up surveys with both groups of participants were conducted 6- and 12-months following reinstatement, asking the same questions about gambling involvement, gambling problems, knowledge, desire to gamble, and help seeking. Each person was emailed a unique code and given a link to complete the survey. An honorarium was provided to the participants in the form of a gift card for non-gambling related merchandise for their participation.

### Measures

The online survey included questions on demographics including age, sex, income, ethnic group, and education level. To measure gambling behaviour, each person was asked about their participation in seven types of gambling including: casino games, slot machines, lotteries, racetrack betting, bingo, sports betting and other games. Frequency of gambling activity for each game was measured on a four-point scale (none = 0; once a month or less = 1; more than once a month = 2; every week = 3). Problem gambling was measured by the Problem Gambling Severity Index (PGSI; (Ferris and Wynne 2001). At baseline (shortly before reinstatement), the PGSI and gambling behaviors were framed in two time periods, prior to self-exclusion and the past 6-months during self-exclusion. For the follow-up surveys, presented 6-months and 12-months after reinstatement, the questions focused on the previous 6-months (following reinstatement).

The remaining questions on the questionnaire (e.g., desire to gamble, knowledge of the tutorial content, help-seeking) were focused on the person’s current state at the baseline survey. Knowledge of the tutorial content was measured using an 18-item true false questionnaire examining knowledge of random chance and erroneous beliefs about gambling, which was tailored specifically to the content of the tutorial. Desire to gamble was measured using five questions on a visual analog scale (Berger et al. 1996; Duncan et al. 2001; Schüll 2013) that we adapted for gambling (e.g., “I would like to gamble”, “Gambling would make my discomfort go away”). Help seeking and self-excluding again were simple yes/no questions.

## Results

Table 2 gives the PGSI scores by sex for baseline and follow-up surveys. For the follow-up surveys, 107 participants provided data at 6-months for a 44% rate of re-contact, and 113 provided data at one year for a 49% re-contact rate. Overall, there was no significant difference between male and females on the PGSI, F (1, 128) = 2.5, *p* < 0.01. There was a significant repeated measures score of time, F (3, 384) = 31.6, *p* < 0.001. There was no interaction between sex and time, F (3, 384) = 0.9, ns. A comparison of PGSI scores from before self-exclusion and during self-exclusion of people who completed the follow-up surveys compared to those who did not found that those who did complete the follow-up data tended to report more problems prior to self-exclusion (M = 10.5) than those who did not complete at least one of the follow-up surveys (M = 7.9), t (233) = 3.25, *p* < 0.001, d = 0.42. There was no difference between survey completers (SD = 5.5, SD = 6.4) and non-completers (M = 5.0, SD = 5.5) of the follow up survey in terms of PGSI scores during self-exclusion, t = -0.63, ns, d = 0.08. Knowledge scores were significantly higher for survey completers (M = 0.82, SD = 0.14) compared to non-completers (M = 0.72, SD = 0.20). Overall, attitude scores did not differ between survey completers and non-completers, but completers rated themselves higher on the final question “I feel I can control my gambling” (SD = 68, SD = 32) than non-completers (M = 58, SD = 33), t = 2.2, *p* < 0.05. In summary, people who completed the follow-up surveys were more problematic gamblers prior to self-exclusion, but had more knowledge and believed they could control their gambling.

**Table 2:** PGSI scores (and standard deviations) before self-exclusion, during self-exclusion, 6-months after reinstatement and 12-months after reinstatement by sex

	Sex	Raw Data			Complete data		
		N	Mean PGSI	SD	N	Mean PGSI	SD
Before self-exclusion	Female	95	9.2	6.4	54	9.7	7.0
	Male	138	9.5	6.3	75	11.2	6.0
During self-exclusion	Female	95	4.9	6.1	54	4.0	5.9
	Male	138	5.6	6.0	75	6.6	6.6
6-month follow-up	Female	44	6.9	6.3	54	6.7	6.1
	Male	63	8.2	6.4	75	7.7	6.3
12-month follow-up	Female	46	6.3	6.1	54	6.7	6.6
	Male	67	7.5	6.5	75	7.5	6.5

Complete data means we used if we only had 6 month follow-up data we used it for the 12-month data and vice versa. One person did not provide their sex

### Gambling Frequency and Gambling Problems

The first hypothesis was that self-exclusion would result in less gambling and lower scores on the PGSI and that this would be sustained at 6 and 12-months. To test this hypothesis, we examined the data on problem gambling. As shown in Table 2, there was a substantial drop in PGSI scores from before self-exclusion, 10.5 (6.2) to during self-exclusion, 5.6 (5.9). PGSI scores at 6-months (M = 7.6; 6.1), t = 5.6, *p* < 0.001 and 12-months (M = 7.1, SD = 6.5), t = 5.6, *p* < 0.001, were significantly lower than before self-exclusion. Two thirds (67.7%) of the sample had a lower score on the PGSI at the 12-month follow-up compared to their PGSI score before self-exclusion. Before self-exclusion 66.4% of the sample scored as severe problem range (8 or more on PGSI) whereas 12-months after reinstatement 38.9% of the sample scored in the severe problem range, z = 4.5, *p* < 0.001. Self-exclusion appears to have resulted in a sustained drop in PGSI scores during the reinstatement follow-up period.

**Fig. 1:**
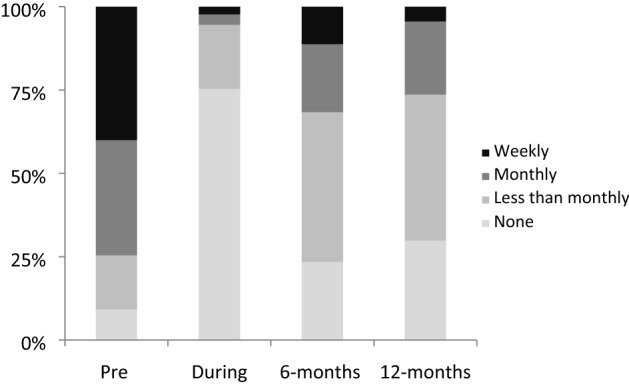
Casino gambling prior to self-exclusion, during self-exclusion, 6-months after reinstatement, and 12-months after reinstatement

**Fig. 2:**
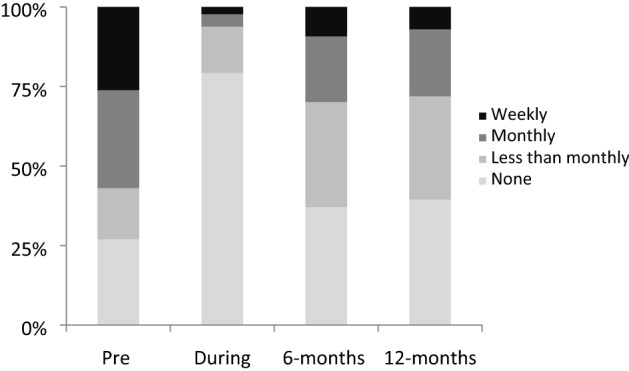
Slot Machine gambling prior to self-exclusion, during self-exclusion, 6-months after reinstatement, and 12-months after reinstatement

Figures 1 and 2, illustrate that a similar pattern occurred with casino, slot machine, and lottery play. Gambling frequency peeked before self-exclusion; decreased during self- exclusion and increased after reinstatement, but did not return to the frequency before self-exclusion. Contrasting gambling before self-exclusion with gambling 12-months after reinstatement, casino gambling, z = -7.2, *p* < 0.001, and slot machine gambling, z = 5.3, *p* < 0.001showed significant decreases. Interestingly, lottery gambling, z = − 3.8, *p* < 0.001 also showed a significant decrease even though self-exclusion in Ontario does not actually cover lottery gambling at bricks and motor retailers. Race track bets, z = 1.3, ns, bingo, z = − 0.3, ns, sports bets, z = − 0.6, ns, and other games, z = 1.6, ns, showed no significant change from before self-exclusion to 12-months after reinstatement. In summary the first hypothesis was confirmed for both gambling problems and gambling frequency.

The second hypothesis was that 6-months and 12-months after being reinstated, people in the experimental (tutorial) condition would be gambling less often and less problematically than those in the control group (no tutorial). The PGSI scores for before self-exclusion, during after self-exclusion, after 6-months, and after 12-months are shown in Table 3. Scores were consistent across the two groups at baseline and throughout the study. A shown in Table 3, the problem gambling scores as measured by the PGSI were significantly higher before self-exclusion than during self-exclusion and at 6 and 12-months in both the control and the tutorial groups. Additionally, the PGSI scores were significantly higher at 6 and 12-months compared to during self-exclusion. In summary, the results suggest that the tutorial did not have any significant impact on problem gambling scores. However, the results do suggest that self-exclusion had a measurable impact on problem gambling. The change from before self-exclusion to during self-exclusion was highly significant in both control and tutorial conditions. This was sustained 12-months after reinstatement of gambling.

**Table 3:** Mean PGSI scores (and Standard deviations) before self-exclusion, during self-exclusion, 6-months after reinstatement and 12-months after reinstatement for the control and the tutorial group

	Tutorial	N	Mean	Std. Deviation
Before self-exclusion	Control	73	10.1	6.6
	Tutorial	57	11.1	6.4
During self-exclusion	Control	73	4.8	5.9
	Tutorial	57	6.4	7.0
6-months after reinstatement	Control	73	7.2	6.2
	Tutorial	57	7.5	6.3
12-months after reinstatement	Control	73	6.7	6.5
	Tutorial	57	7.6	6.6

The main effect of test time (before self-exclusion, during self-exclusion, 6-month after reinstatement, 12-months after reinstatement) was significant, F (3, 384) = 31.4, *p* < 0.001, the main effect of tutorial vs. control was not significant, F (1, 128) = 1.5 ns, and the crucial interaction was not significant, F (3,384) = 0.5, ns, indicating that the control and experimental conditions did not differ over time.

**Fig. 3:**
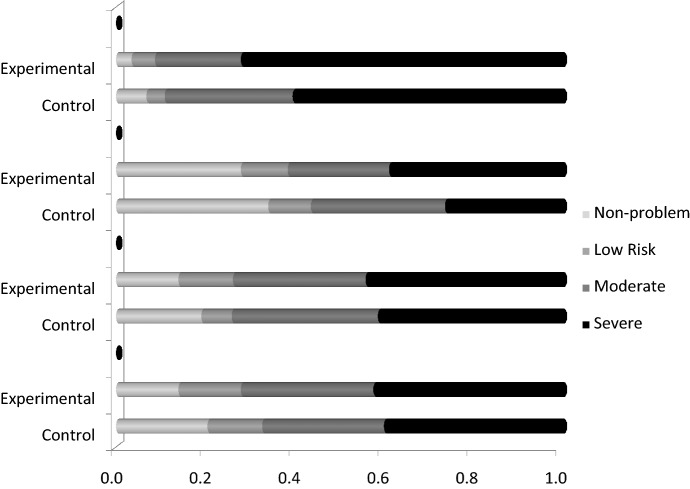
Problem gambling severity index categories by experimental group

This change is also graphically depicted in Fig. 3 illustrating the shift in severe problem gamblers from before self-exclusion to the follow-up surveys. Overall, 65.4% of the sample reported lower PGSI scores at 6-months after reinstatement. The decrease for the experimental group was somewhat larger (71.9%) than the control group (60.3%), but was not significantly different, chi-square = 1.9, ns. The effect was estimated as a correlation of r = 12. A power analysis indicated that it would require a sample size of 543 for an 80% power to reach significance. An analysis of the 12-month data indicated that 67.7% of the sample had lower PGSI scores after 6-months. There was no difference between the control (68.5%) and the experimental group (66.7%).

We also examined the correlations between PGSI scores across the four time periods. As shown in Table 4, the correlation in PGSI scores across the four time periods was moderately strong, but the strongest correlation was between the 6-month and 12-month follow up data, r = 0.73, *p* < 0.001. The weakest correlations were between the PGSI scores during self-exclusion and the 12-month follow-up. This last finding is important because it suggests that PGSI scores before and during self-exclusion are not strong indicators of PGSI scores one year after reinstatement, accounting for 15% and 13% of the variance of problem severity after 12-months respectively.

**Table 4:** Correlations of PGSI scores across the 4 time periods and help seeking behaviors during the follow-up

	Help seeking/Self-exclusion		PGSI		
	6-mths	12-mths	Before	During	6-mths
Help seeking/self-exclusion 12-months	0.64*** n = 92				
PGSI Before	0.29** n = 104	0.07 n = 114			
PGSI During	0.18 n = 104	0.00 n = 114	0.61*** n = 130		
PGSI 6-months	0.41*** n = 104	0.30** n = 91	0.51*** n = 107	0.60*** n = 107	
PGSI 12-months	0.34** n = 91	0.41*** n = 113	0.39*** n = 113	0.37*** n = 113	0.73*** n = 90

*p < .05; **p < .01; ***p < .001

### Games Played

As noted above, gambling decreased significantly from before self-exclusion to during self-exclusion for slots, casino games, and lottery products. An examination of the individual games found the same pattern of decreases in gambling for casino games, slot machines and lotteries for both tutorial and control group. An examination of each of the 7 types of gambling found no evidence of an impact of the tutorial on game frequency (see Supplementary Tables 2 and 3). An examination of the individual games found the same decreases in gambling for casino games, slot machines and lotteries for both tutorial and control group and no evidence of an interaction between experimental condition over time. To simplify the analysis an aggregate of games frequencies was computed by adding the individual game scores together. The within subjects variable was highly significant, F (3, 384) = 76.8, *p* < 0.001. The difference between control and tutorial groups was not significant, F (1, 128) = 0.0, ns, and the interaction was not significant, F (3, 384) = 1.9, ns.

### Help Seeking and Self-excluding

The third hypothesis (H3) was that 6-months and12-months after being reinstated people in the experimental group would be more likely to seek treatment or return to self-exclusion than those in the control condition. At 6-months and 12-months people were asked if they have sought help and if they had self-excluded again. Only a minority of the participants sought help or self-excluded again during the 12-month follow up period. A total of 13% of the sample sought help at both 6-months and 18% sought help at 12-months for a combined total of 19% who sought help or self-exclusion during that period. Similarly, 20% of the sample sought self-exclusion at 6-months and 17% sought self-exclusion at 12-months for a combined it 21% who sought help or self-exclusion during that period. Helping seeking and self-exclusion were correlated at both 6-months, r = 0.40, *p* < 0.001, and 12-months, r = 0.59, *p* < 0.001 and when across the two time periods, r = 0.69, *p* < 0.001. Due to the small number of people who sought help or self-exclusion, we combined them together into a single help-seeking or self-exclusion variable. Slightly more of the participants in the tutorial group sought out help or self-excluded than the control group at both 6-months and (32 vs 25%) and at 12-months (25 vs. 17%), and when the two follow-up survey were combined (30% vs. 25%). However, none of these contrasts reached significance, e.g., chi-square (1) = 0.43, ns.. We estimated an effect size of r = 0.10 between group (control vs. tutorial) and the combined help or self-exclusion (no vs yes) of r = 0.10. A power analysis based on this correlation indicated that it would require a sample of approximately 800 participants in total to have an 80% chance of the effect reaching significance.

Help seeking and self-exclusion were correlated with PGSI scores. People who had higher PGSI scores before self-exclusion were more likely to seek help or self-exclude again after 6-months, r = 0.29, *p* < 0.001, but this link disappeared by 12-months. The strongest relationship between PGSI scores and help seeking or self-exclusion was during the same time period. That is PGSI scores at 6-months were most strongly related to help seeking or self-exclusion at 6-month, r = 0.41, *p* < 0.001, and PGSI scores at 12-months were most strongly related to help seeking or self-exclusion at 12-month, *p* < 0.41, *p* < 0.001. A logistic regression predicting help seeking at the various time periods found that only problems at 6-months predicted help-seeking at 6-months, and that only problems at 12-months predicted help seeking at 12-months.

As noted above 38.9% people still fell into the severe range of the PGSI after 12-months. However, considering help seeking and self-exclusion together, the number of severe problem gamblers still actively engaging in gambling dropped to 27.7%.

### Knowledge

The fourth hypothesis was that at 6-months and at 12-months after being reinstated, people in the experimental condition will have higher scores on the knowledge test than those in the control group. The surveys asked 18 questions about knowledge of gambling and probability based on the content of the tutorial. Two questions were dropped because the answer options were ambiguous. For example, “you have to bet big to win big is” is true but it can be a poor choice because it can also lead to larger losses. The remaining 16 were scored as correct (1) or incorrect (0) and the total was computed. The Cronbach alpha for the 16-item scale was 0.75 indicating adequate reliability. The results are shown in Table 5. Participants correctly answered a little more than 80% of the questions. The tutorial group scored a little higher than the control group at all three time periods including baseline, but this was not significant. There was a significant within-subject effect, F (2, 262) = 6.6, *p* < 05, but the comparison of control and tutorial groups was not significant, F (1, 131) = 2.4, ns, and the interaction was not significant, F (1, 131) = 2.4, ns and F (1, 262) = 0.8, ns. An examination of contrasts indicated that the difference between baseline and 6-month follow-up was significant, t (132) = 3.6, *p* < 0.001, d = 0.33. The contrast between baseline and 12- month follow-up was not significant after controlling for multiple comparisons, t (132) = -0.22, ns, d = 0.02. The relationship between group membership and knowledge scores at 6-months was *r* = 0.15 which would require a sample of 350 participants for an 80% power to reach significance and at 12-months, *r* = 0.06, which would require a sample of about 2200 participants for an 80% power to reach significance.

**Table 5:** Average score on knowledge items

	Group	N	Mean (%)	Std. deviation (%)
At Baseline	Control	73	80	14
	Tutorial	60	83	14
6-months After reinstatement	Control	73	83	13
	Tutorial	60	87	12
12-months After reinstatement	Control	73	83	13
	Tutorial	60	85	14

Contrast of baseline vs 6-months is significant, but there was no interaction

### Desire to Gamble

The final hypothesis (H5), was that at 6- and 12-months after being reinstated people in the experimental group will be less likely to express a strong desire to gamble than those in the control group. There were five questions on the survey used to measure participants’ desire to gamble. All were measured with a sliding scale from 0 to 100. As shown in Table 6, the means for control vs. tutorial analysis were not significant for all five questions on desire to gamble questions, nor was there an interaction for any of the five items. In fact, there were no significant contrasts between control vs. tutorial. The only effects that did reach significance were within subject effect for “I intend to gamble in the near future”, F (2,262) = 4.0, *p* < 0.05, and “I feel I can control my gambling” F (2,262) = 8.5, *p* < 0.001 both of which decreased from baseline to 6 months.

**Table 6:** Means of attitude questions (desire to gamble)

			N	Reinstatement		6-months		12-months	
1. Like to gamble:		C	73	34.6	28.6	33.4	31.0	35.9	32.5
		T	60	36.3	29.6	39.8	32.0	40.1	31.2
2. Intend to gamble		C	73	42.9	33.3	33.7	31.9	33.9	33.2
		T	60	43.2	33.3	37.8	33.6	38.8	35.5
3. Feel better		C	73	15.4	24.4	14.9	24.2	13.6	21.8
		T	60	15.2	21.8	14.2	23.5	13.2	23.5
4. Rid discomfort		C	73	11.5	23.1	10.8	24.2	9.1	22.0
		T	60	11.4	22.1	11.1	23.3	8.7	21.1
5. Can control		C	73	67.7	34.2	58.5	35.2	59.4	35.8
		T	60	68.0	32.8	49.5	35.5	55.8	35.8

Missing values in the follow up data were dealt with in the same way as with PGSI scores. Note: 1. = I would like to gamble; 2. = I intend to gamble in the near future; 3. = Gambling will make me feel better; 4. = Gambling will get rid of my discomfort; 5. = I feel I can control my gambling. C = Control; T = Tutorial

Table 7 presents correlations of each of these “desire to gamble” questions with PGSI scores, knowledge scores, and help-seeking and self-exclusion, each correlation is within its own time period (e.g., 6 intending the gamble at 6-months and PGSI scores at 6 months). Higher scores on the first four items was positively associated with PGSI scores, and negatively associated with knowledge scores. “Control” however showed the opposite pattern. Feeling in control was negatively related to problem gambling scores at 6-months, r = -0.56, *p* < 0.001, and 12-months, r = -0.47, *p* < 0.001, but not at baseline, suggesting that after 6 month after reinstatement, some people who feel in control, are in control of their gambling. For knowledge scores, the strongest relationship was with “gambling would get rid of discomfort”, *r* = 0.50, *p* < 0.001 at baseline but this relationship was not significant at 6 or 12-months. Intending on gambling and feeling in “control” at 6 and 12-months were negative related to help-seeking or self-exclusion. We also examined the correlation across time periods. “I can control my gambling” at 6 months was negatively associated with help-seeking or exclusion at 12 months, r = -0.39, *p* < 0.001.

**Table 7:** Correlations with questions on the desire to gamble

	Like to gamble	Intend to gamble	Would feel better	Get rid of discomfort	Can control gambling
Knowledge and desire at baseline (N = 256)	− .21**	− .05	− .43**	− .50^**^	.20**
Knowledge and desire 6-months (N = 104)	.16	.21*	− .03	-.13	.29**
Knowledge and desire at 12-months (N = 114)	.00	− .02	− .05	− .07	.13
PGSI Before and desire (N = 256)	.32**	.22**	.28**	.28**	− .11
PGSI During and desire (N = 256)	.32**	.22**	.34**	.36**	− .17**
PGSI and desire at 6-month (n = 104)	.22**	.08	.26**	.31**	− .56**
PGSI and desire at 12-month (N = 114)	.45**	.04	.17	.25**	− .47**
Helping seeking or self-exclusion at 6 months	− .10	− .27**	− .09	.01	− .26**
Helping seeking or self-exclusion at 12 months	.14	− .08	.06	.04	− .25**

In each case the correlations are for the desire items and other variables within the same time period; that is “intend to gamble” at 6-month with knowledge scores at 6-months. Before = Before self-exclusion and During = During self-exclusion

*p < .05; **p < .01

## Discussion

The purpose of this study was to examine reinstatement and to evaluate the effectiveness of a reinstatement tutorial video in reducing gamblers re-engaging in problematic gambling after a period of self-exclusion. This project was guided by five hypotheses. The only hypothesis that was confirmed was the first one: the self-exclusion program itself would reduce problem gambling. In addition, this reduction was sustained at the 6- and 12-months follow-up surveys after reinstatement. We found no significant evidence that the tutorial reduced problem gambling scores or gambling behavior for the tutorial group more than for the control group. Similarly, we found that help-seeking, desire to gamble and knowledge of random chance were not significantly changed by completing the video tutorial. A few variables trended in the predicted direction, but power analyses suggest that the sample size would have to substantially larger for this study to reach significance. Future evaluation studies of reinstatement tutorials need to assume a small effect size and draw a much larger sample.

Previous research on self-exclusion has focused on self-exclusion itself as an intervention. The results have been mixed in part because self-exclusion is not consistently enforced by the gambling industry. Self-excluders generally experience benefits from self-exclusion programs (Gainsbury 2014) including a decrease in their gambling and an improvement in their psychological well-being in the short-term. However according to Gainsbury (2014), the benefits of self-exclusion may decline over time. The current study found that the benefits of self-exclusion were sustained for 12-months.

Gainsbury (2014) suggests caution regarding a causal relationship between self-exclusion and a decrease in gambling behaviour and/or gambling harms. There is no evidence that self-exclusion causes a reduction in gambling (Gainsbury 2014). The gamblers have already demonstrated a willingness to address their gambling simply by applying for self-exclusion (Gainsbury 2014). Nonetheless, self-exclusion does appear to be helpful for some people. A recent study by Pickering, et al., argues that gambling operators should increase marketing efforts to promote self-exclusion programs to their patrons (Pickering et al. 2019). In addition, they suggest streamlining the registration processes to make the system easier to enter a self-exclusion program as well as training staff and using technology in order to help identify patrons who might need a self-exclusion program.

Gainsbury (2014) recommends that self-excluders whose exclusion term has ended be contacted prior to the term of their self-exclusion and provided with information about treatment services and referrals to treatment services. Nowatzki and Williams also recommend having educational information provided to people who reinstate (Nowatzki and Williams 2002). To the best of our knowledge, this study is the first that evaluated the efficacy of a reinstatement tutorial following a period of self-exclusion. This is also the first study that we know of that has longitudinally followed a group of people who reinstated after self-exclusion. This provided us with an opportunity to determine if self-exclusion has any long-term effect. In addition, as far as we know this is the first controlled study to examine the efficacy of an intervention aimed at reducing the harm of problem gamblers. Given that the participants were reinstating after self-exclusion, it is not surprising that their gambling increased from the period of self-exclusion. However, it is encouraging that they did not return to gambling as often as they had prior to self-exclusion. In fact, most of the participants (in both tutorial and control group) reported less gambling and less problem gambling during the follow-up period than prior to self-exclusion. In addition, they showed a substantial reduction in PGSI scores from their pre-exclusion gambling to their post reinstatement gambling. These results suggest that most of the participants reduced their gambling as a result of self-exclusion and that this decrease in gambling was sustained for many of the participants in the study after reinstatement. Moreover, there was no evidence that the participants increased other types of gambling as a substitution either during self-excluding or after reinstatement. Thus, self-exclusion appears to be an effective way of decreasing gambling. However, 40% of the participants reported gambling problematically 12-months after reinstatement. However, if we excluded those who choose to seek out help or self-excluded again, this figure dropped to 27%. Nonetheless, greater efforts need to be made to continue encouraging responsible play.

As noted above, there was no evidence found for an effect of the tutorial on the frequency of play or number of problems, however there were a number of other interesting finds. For example, help seeking was rare among the participants, demonstrated by the minority of participants who sought help or self-excluded again during the 12-month follow up period. The results of the logistic regression suggest that help seeking was driven by immediate problems and not by persistent problems. However, when “can control” gambling at 6-months was added to the model predicting help seeking or self-exclusion at 12-months it had a significant negative effect and PGSI scores at 12-months dropped to non-significance. It would seem that recognition that one cannot control one’s gambling is the best predictor of future help seeking or self-exclusion actions.

Results of the final desire to gamble item, “can control”, produced some important results. First, scores on this variable were generally higher than the average of the other desire questions suggesting that most of the participants believe they can control their gambling. However, “can control” showed a significant decrease from baseline survey to 12-month survey. In addition, scores on “can control” at baseline were not significantly correlated with PGSI scores, however at the 6-month and 12-month surveys, “can control” produced fairly strong negative correlations with PGSI scores. These two findings suggest that the participants have become more aware of their inability to control their own problem and have become more honest with themselves. In addition, this greater honesty was associated with a greater chance of seeking help or self-excluding at 12-months. Another interesting finding was that “getting rid of discomfort” was significantly associated with PGSI scores at baseline, 6-months, and 12-months. This finding would suggest that relief from pain or discomfort may be an important factor in problem gambling (Barry et al. 2013).

In this study, we examined the efficacy of providing more resources at reinstatement. The overall results suggest that the reinstatement tutorial did not have any significant impact on problem gambling, desire to gamble, or knowledge about random chance. Perhaps the reason for this is that in most of the larger casinos in Ontario, both tutorial and control group already had access to PlaySmart Centres. As noted before, part of the reasons for introducing a mandatory tutorial is that most patrons of the casino do not use the PlaySmart Centres.

Perhaps a more likely explanation is that an information based intervention is simply not sufficient to prevent a relapse. Problem gambling is viewed as a behavioral addiction (American Psychiatric Association 2013): A set of habit patterns that are initiated outside of consciousness (see Marlatt and Gordon 1985). Information-only approaches for relapse prevention may have at best only a limited impact (see Marlatt and Gordon 1985). The findings suggest that stronger relapse prevention methods are needed to help people become more resistant to problem gambling relapse.

We applaud the OLG for adding this educational video tutorial as a requirement to the reinstatement process. Perhaps some sort of brief counselling such as motivational interviewing or lessons in mindfulness might be a better option than a purely informational tutorial. In keeping with the spirit of the original tutorial, and given the current COVID-19 pandemic, such interventions could be offered on-line (van der Maas et al. 2019).

This study did find that self-exclusion is beneficial to gamblers in helping them manage their gambling and decreasing problem gambling on a long-term basis. Gambling venues should offer and advertise self-exclusion programs for gambling patrons as a harm reduction measure to enhance responsible gambling practices.

## Limitations

A limitation of the study is that the results are all based on self-report data. However, the internal consistency of the results across the three questionnaires suggests that people are taking the questions seriously and trying to answer accurately. A second limitation is the attrition rate of 50%. This attrition rate is somewhat high, but that is likely because we only had one way of contacting the participants (email) and email messages may have been classed as junk mail by servers or missed due to the huge flood of junk email that often overwhelms email accounts. The attrition rate in the control and experimental group was similar. Finally, our sample was a convenience sample of gamblers who chose to self-exclude during the time period; that is, we were not able to randomly assign the participants to tutorial and control.

## Conclusion

In summary, during self-exclusion, people showed a substantial decrease in gambling problems and gambling behavior. After reinstatement, their gambling increased, but did not return to the levels reported prior to self-exclusion. The results suggest that self-exclusion can be beneficial to some people to help them reduce the harm of gambling. Previous studies (Gainsbury 2014; Nowatzki and Williams 2002) have recommended that self-excluders who wish to be reinstated should first be provided with mandatory educational resources and to possibly receive referral to treatment services as well. Until now, no research has been conducted on the efficacy of reinstatement education. The current paper evaluated the efficacy of a mandatory tutorial and found no significant impact on gambling, gambling problems, knowledge of gambling, desire to gamble or on help seeking. The tutorial group was non-significantly more likely to seek help than the control group, but the observed effect size ranged from small to very small suggesting future evaluation studies need to have the power to detect a small effect size. The lack of significant findings may suggest that a purely information based approach may not be effective at helping problem gamblers avoid relapse. In conclusion, we found a substantial reduction in harm up to 12-months after reinstatement from self-exclusion, however, we found no significant additional harm reduction resulting from the tutorial intervention. More work is needed on improving the outcome of self-exclusion programs.

## Supplementary Information

Supplementary file1 (DOCX 44 kb)
