# Supplementary material for: Efficacy of a Voluntary Self-exclusion Reinstatement Tutorial for Problem Gamblers
Source: J Gambl Stud. 2021 Jan 23;37(4):1245–62. doi: 10.1007/s10899-021-09998-x (PMC7825392; doi:10.1007/s10899-021-09998-x)
Supplement: Supplementary file 1 — Supplementary file1 (DOCX 44 kb) [file 10899_2021_9998_MOESM1_ESM.docx]

**Supplementary text**

**Tutorial content**

Table S1 provides a description of the content of the video tutorial that was required to people in the Tutorial group.

**Changes in types of gambling**

Prior to self-exclusion, 40% reported gambling as a casino between once and month and once a week and 35% reported gambling at a casino weekly or more often. In addition, prior to self-exclusion, 31% reported gambling on a slot machine between once a month and once a week and 26% reported gambling on a slot machine weekly or more often. During self-exclusion, most of the participants reduced their gambling substantially with 75% not gambling at all at a casino and 79% reporting not playing on a slot machine. During self-exclusion only 5% reported casino gambling and 7% reported slot machine gambling, monthly or more often. At 6 and 12-months, both casino and slot machine gambling had increased compared to during self-exclusion, but was still substantially lower than before self-exclusion. Interestingly lottery purchases also decreased during self-exclusion and remained lower after reinstatement even though lotteries were not included in the self-exclusion agreement. We also examined the data for a possible substitution effect where after having banned themselves from casinos and slot machine venues they would increase their plan on other games. There was no evidence of a substitution effect (see Supplementation Table S2).

As shown in Table S3, participants reported substantial decreases in gambling on slots and casino from before self-exclusion to during self-exclusion on casino games, z= -9.3, p< .001, slot machines, z= -8.0, p< .001, and lotteries, z= -4.9, p< .001. No significant change was reported on horse race betting, bingo, sports betting or other games. Similarly comparing gaming before self-exclusion to the game participation 6-months later, showed a substantial decrease in participation in casino games, z= -5.5, p< .001, slot machines, z= -3.1, p< .01, and lotteries, z= -2.6, p< .01, and again, no change was found for horse race betting, bingo, sports betting or other games. The 12-months data was very similar to the 6-month data with significant decreases for casino games, z= -7.2, p< .001, slot machines, z= -5.3, p< .01, and lotteries, z= -3.8, p< .01, and again, no change was found for horse race betting, bingo, sports betting or other games.

We then examined the data comparing gambling during self-exclusion to gambling after reinstatement. There was a substantial increase in participation in casino games, z= 5.8, p< .001, and slot machines, z= -5.1, p< .001, but no change was found for lotteries, horse race betting, bingo, sports betting or other games. The same results were found for the 12-month data.

Supplementary Table S1

Reinstatement Tutorial Video Content

- Information about how slot machines and other games work.
- Encouraging customers know their limits.
- Information on the odds of winning.
- A cost of play calculator.
- Importance of setting a budget based on what one can afford to lose.
- Why chasing is a bad idea.
- Explains why even on a skill games one can lose.
- Outlines some of the more common red flags to help them gamble safely.
- Information on gambling facts from gambling myths.
- High risk and lower risk ways to gamble.
- The importance of playing for entertainment.
- Information on gambling counselling.
- A quiz to help people understand the games better.

Supplementary Table S2

Details of play on all 7 types of gambling for each time period.

| Prior to self –exclusion | | |  |  |  |  |  |
| --- | --- | --- | --- | --- | --- | --- | --- |
| X | Casino | Slots | lottery | race | Bingo | sports | Other |
| None | 9.2 | 26.9 | 20.0 | 90.8 | 80.0 | 76.9 | 77.7 |
| Less than monthly | 16.2 | 16.2 | 30.0 | 6.9 | 15.4 | 12.3 | 3.1 |
| Monthly | 34.6 | 30.8 | 25.4 | 1.5 | 3.8 | 6.2 | 5.4 |
| Weekly | 40.0 | 26.1 | 24.6 | 0.8 | 0.8 | 3.8 | 4.6 |
|  |  |  |  |  |  |  |  |
| During Self Exclusion | | |  |  |  |  |  |
| X | Casino | Slots | lottery | race | Bingo | sports | Other |
| None | 75.4 | 79.2 | 36.2 | 95.4 | 83.8 | 81.5 | 80.8 |
| Less than monthly | 19.2 | 14.6 | 29.2 | 2.3 | 9.2 | 10.0 | 4.6 |
| Monthly | 3.1 | 3.8 | 18.5 | 1.5 | 5.4 | 3.8 | 2.3 |
| Weekly | 2.3 | 2.3 | 16.2 | 0.0 | 1.5 | 4.6 | 3.1 |
|  |  |  |  |  |  |  |  |
| 6-months after Reinstatement | | |  |  |  |  |  |
| X | Casino | Slots | lottery | race | Bingo | sports | Other |
| None | 23.5 | 36.7 | 33.7 | 96.9 | 84.7 | 82.6 | 86.7 |
| Less than monthly | 44.9 | 32.7 | 29.6 | 1.0 | 7.1 | 8.2 | 7.1 |
| Monthly | 20.4 | 20.4 | 21.4 | 1.0 | 7.1 | 7.1 | 2.0 |
| Weekly | 11.2 | 9.2 | 15.3 | 1.0 | 1.0 | 2.0 | 4.1 |
|  |  |  |  |  |  |  |  |
| 12-months after Reinstatement | | | |  |  |  |  |
| x | Casino | Slots | lottery | race | Bingo | sports | Other |
| None | 29.8 | 39.5 | 30.7 | 94.7 | 80.7 | 76.3 | 79.8 |
| Less than monthly | 43.9 | 32.5 | 28.1 | 4.4 | 7.9 | 9.7 | 7.0 |
| Monthly | 21.9 | 21.1 | 31.6 | 0.9 | 7.9 | 6.1 | 2.6 |
| Weekly | 4.4 | 7.0 | 9.7 | 0.0 | 1.8 | 7.0 | 2.6 |

Note: percentages do not add up to 100% because some people did not respond, especially for other.

Supplementary Table S3

Control group game frequency

| Before | Casino | Slots | Lottery | Race | Bingo | Sports | Other |
| --- | --- | --- | --- | --- | --- | --- | --- |
| None | 8 | 38 | 23 | 92 | 75 | 78 | 81 |
| Less than monthly | 16 | 15 | 29 | 7 | 18 | 10 | 3 |
| Monthly | 34 | 25 | 22 | 1 | 5 | 4 | 7 |
| Weekly | 41 | 22 | 26 |  | 1 | 7 | 4 |
|  |  |  |  |  |  |  |  |
| During | Casino | Slots | Lottery | Race | Bingo | Sports | Other |
| None | 71 | 77 | 40 | 96 | 85 | 82 | 84 |
| Less than monthly | 22 | 15 | 26 |  | 5 | 8 | 4 |
| Monthly | 4 | 7 | 16 | 3 | 7 | 4 | 3 |
| Weekly | 3 | 1 | 18 |  | 3 | 5 | 4 |
|  |  |  |  |  |  |  |  |
| 6-months | Casino | Slots | Lottery | Race | Bingo | Sports | Other |
| None | 25 | 47 | 30 | 95 | 83 | 81 | 90 |
| Less than monthly | 46 | 29 | 37 | 2 | 8 | 6 | 7 |
| Monthly | 17 | 19 | 19 | 2 | 8 | 10 | 2 |
| Weekly | 11 | 5 | 14 | 2 | 2 | 3 | 2 |
|  |  |  |  |  |  |  |  |
| 12-months | Casino | Slots | Lottery | Race | Bingo | Sports | Other |
| None | 36 | 49 | 30 | 93 | 79 | 79 | 86 |
| Less than monthly | 36 | 25 | 31 | 5 | 8 | 7 | 9 |
| Monthly | 23 | 23 | 31 | 2 | 10 | 8 | 2 |
| Weekly | 5 | 3 | 8 |  | 3 | 7 | 4 |

Note: percentages do not add up to 100% because some people did not respond, especially for other.

Supplementary Table 2 continued

Tutorial group game frequency

| Before | Casino | Slots | Lottery | Race | Bingo | Sports | Other |
| --- | --- | --- | --- | --- | --- | --- | --- |
| None | 11 | 12 | 16 | 89 | 86 | 75 | 86 |
| Less than monthly | 16 | 18 | 32 | 7 | 12 | 16 | 4 |
| Monthly | 35 | 39 | 30 | 2 | 2 | 9 | 4 |
| Weekly | 39 | 32 | 23 | 2 |  |  | 6 |
|  |  |  |  |  |  |  |  |
| During | Casino | Slots | Lottery | Race | Bingo | Sports | Other |
| None | 81 | 82 | 32 | 95 | 82 | 81 | 90 |
| Less than monthly | 16 | 14 | 33 | 5 | 14 | 12 | 6 |
| Monthly | 2 | 4 | 21 |  | 4 | 4 | 2 |
| Weekly | 2 |  | 14 |  |  | 4 | 2 |
|  |  |  |  |  |  |  |  |
| 6-months | Casino | Slots | Lottery | Race | Bingo | Sports | Other |
| None | 21 | 21 | 41 | 100 | 88 | 85 | 85 |
| Less than monthly | 44 | 41 | 18 |  | 6 | 12 | 8 |
| Monthly | 24 | 21 | 26 |  | 6 | 3 | 3 |
| Weekly | 12 | 18 | 15 |  |  |  | 5 |
|  |  |  |  |  |  |  |  |
| 12-months | Casino | Slots | Lottery | Race | Bingo | Sports | Other |
| None | 23 | 28 | 32 | 96 | 86 | 75 | 88 |
| Less than monthly | 53 | 42 | 25 | 4 | 8 | 13 | 6 |
| Monthly | 21 | 19 | 32 |  | 6 | 4 | 4 |
| Weekly | 4 | 11 | 11 |  |  | 8 | 2 |

Note: percentages do not add up to 100% because some people did not respond, especially for other.
